# The microbiome dynamics and interaction of endosymbiotic Symbiodiniaceae and fungi are associated with thermal bleaching susceptibility of coral holobionts

**DOI:** 10.1128/aem.01939-23

**Published:** 2024-03-06

**Authors:** Biao Chen, Yuxin Wei, Kefu Yu, Yanting Liang, Xiaopeng Yu, Zhiheng Liao, Zhenjun Qin, Lijia Xu, Zeming Bao

**Affiliations:** 1 Guangxi Laboratory on the Study of Coral Reefs in the South China Sea, Coral Reef Research Center of China, School of Marine Sciences, Guangxi University, Nanning, China; 2 Southern Marine Science and Engineering Guangdong Laboratory (Guangzhou), Guangzhou, China; 3 Key Laboratory of Environmental Change and Resource Use in Beibu Gulf, Ministry of Education, Nanning Normal University, Nanning, China; 4 South China Institute of Environmental Sciences, MEE, Guangzhou, China; University of Delaware, Lewes, Delaware, USA

**Keywords:** coral holobiont, microbiome dynamics, potential interaction, Symbiodiniaceae, fungi, thermal bleaching susceptibility

## Abstract

The thermal bleaching percentage of coral holobionts shows interspecific differences under heat-stress conditions, which are closely related to the coral-associated microbiome. However, the ecological effects of community dynamics and interactions between Symbiodiniaceae and fungi on coral thermal bleaching susceptibility remain unclear. In this study, we analyzed the diversity, community structure, functions, and potential interaction of Symbiodiniaceae and fungi among 18 coral species from a high thermal bleaching risk atoll using next-generation sequencing. The results showed that heat-tolerant C3u sub-clade and *Durusdinium* dominated the Symbiodiniaceae community of corals and that there were no core amplicon sequence variants in the coral-associated fungal community. Fungal richness and the abundance of confirmed functional animal-plant pathogens were significantly positively correlated with the coral thermal bleaching percentage. Fungal indicators, including Didymellaceae, Chaetomiaceae, *Schizophyllum*, and *Colletotrichum*, were identified in corals. Each coral species had a complex Symbiodiniaceae–fungi interaction network (SFIN), which was driven by the dominant Symbiodiniaceae sub-clades. The SFINs of coral holobionts with low thermal bleaching susceptibility exhibited low complexity and high betweenness centrality. These results indicate that the extra heat tolerance of coral in Huangyan Island may be linked to the high abundance of heat-tolerant Symbiodiniaceae. Fungal communities have high interspecific flexibility, and the increase of fungal diversity and pathogen abundance was correlated with higher thermal bleaching susceptibility of corals. Moreover, fungal indicators were associated with the degrees of coral thermal bleaching susceptibility, including both high and intermediate levels. The topological properties of SFINs suggest that heat-tolerant coral have limited fungal parasitism and strong microbial network resilience.

**IMPORTANCE:**

Global warming and enhanced marine heatwaves have led to a rapid decline in coral reef ecosystems worldwide. Several studies have focused on the impact of coral-associated microbiomes on thermal bleaching susceptibility in corals; however, the ecological functions and interactions between Symbiodiniaceae and fungi remain unclear. We investigated the microbiome dynamics and potential interactions of Symbiodiniaceae and fungi among 18 coral species in Huangyan Island. Our study found that the Symbiodiniaceae community of corals was mainly composed of heat-tolerant C3u sub-clade and *Durusdinium*. The increase in fungal diversity and pathogen abundance has close associations with higher coral thermal bleaching susceptibility. We first constructed an interaction network between Symbiodiniaceae and fungi in corals, which indicated that restricting fungal parasitism and strong interaction network resilience would promote heat acclimatization of corals. Accordingly, this study provides insights into the role of microorganisms and their interaction as drivers of interspecific differences in coral thermal bleaching.

## INTRODUCTION

Coral reefs are biodiversity hotspots with the most diverse symbioses in the ocean and provide habitats for more than 30% of the marine multicellular organisms (1–3). However, global warming and its amplified marine heatwaves have led to a serious degradation of coral reef ecosystems worldwide (4–6), and global coral cover has dramatically declined by approximately 50%–80% since the 1970s (7). The exceptional marine heatwaves induced by El Niño between 2015 and 2017 directly led to 91.1% of reefs along the Great Barrier Reef (GBR) experiencing thermal bleaching (8), and coral species with staghorn and tabular shapes almost died out (9, 10). This unprecedented long-term tropical marine heatwave has caused an 89% loss of reef-building coral cover in the reefs of Kiritimati in the equatorial Pacific, which has also resulted in large-scale bleaching and high mortality of coral (6, 11). The global climate model predicts that the frequency and scale of coral thermal bleaching events will increase (12, 13) and that annual bleaching events will occur globally in coral reefs from the mid-21st century (14). Intensifying global warming and climate change have led to the collapse of the structure and ecological functions of coral reef ecosystems, resulting in irreversible regime shifts (4, 11). Interestingly, corals showed significant interspecific differences in bleaching severity and heat tolerance during heatwave events. Some coral species occurred bleached at a colony scale in low-temperature conditions (<10% of all coral bleaching), whereas colonies of other species experienced bleaching in extremely high-temperature conditions (>80% of all coral bleaching; e.g., *Pocillopora*, *Acanthastrea*, *Galaxea*, and Fungiidae), which have been found in the Red Sea and GBR (4, 15). However, the reasons for the distinct survival rates of different coral species at extremely high temperatures remain unclear.

Corals are holobionts composed of animal hosts, endosymbiotic Symbiodiniaceae, bacteria, archaea, fungi, and viruses (3, 16, 17). The difference in phenotype, genetic characteristics, transcription, and metabolism of host were closely associated with the environmental adaptability of coral species (18–21); however, coral-associated microbiome also played a crucial role in regulating the environmental tolerance of coral holobiont. Thus, the environmental adaptability of corals is regulated by both the host and its associated microbiome (3, 22, 23), which explains the interspecific differences in heat tolerance and bleaching susceptibility (24–28). The endosymbiotic Symbiodiniaceae are the primary photosymbionts of coral species that play an important role in the health and thermal adaptive potential of coral holobionts (29). Resilient and heat-tolerant Symbiodiniaceae (e.g., *Durusdinium*) can provide additional thermal tolerance to coral holobionts (30, 31). It has been found that *Platygyra ryukyuensis* and *Favites pentagona* initially changed the heat-sensitive dominant Symbiodiniaceae to heat-tolerant *Durusdinium* during long-term marine heatwave events in the El Niño core zone and improved the thermal bleaching resistance of coral holobionts (6). The biogeographic patterns and diversity of Symbiodiniaceae have been widely reported in coral reefs and communities in subtropical and tropical zones (31–36). However, studies on the potential interactions between Symbiodiniaceae and other microbes are rare, and the effects of these interactions on the thermal bleaching susceptibility of distinct coral species have not been accurately assessed. Few studies have focused on the rare symbiont biosphere and interactions between Symbiodiniaceae members. This suggests that rare symbionts will enhance the stability and resistance of coral–Symbiodiniaceae symbioses, allowing them to better respond to external disturbances (37). It has also been found that parasitic symbionts (C7 sub-clade) are inhibited by the dominant *Durusdinium trenchii* in the symbiont interaction of corals in the South China Sea (SCS) (38). In addition, fungi have diverse functions and play key roles in bioerosion, pathogens, biogeochemical cycles, and microbiome structuring in coral reef ecosystems and have the ability to exhibit numerous functions in pelagic and benthic communities (39). Although fungi are important eukaryotic microbes in the coral-associated microbiome and are involved in phosphorus metabolism in holobionts, their diversity, ecology, evolution, and function are not well understood (3, 40, 41). Previous studies have found that fungal communities are extremely heterogeneous and phylogenetically diverse, and no biogeographical or host-specific patterns of coral-associated fungal communities have been accurately described (28, 42, 43). However, coral holobionts show greater diversity and dissimilarity in fungal communities when they exhibit tissue lesions or live in thermal environments (44, 45). The relative abundances of Saccharomycetes and Malasseziomycetes increased in thermally bleached or heat-stressed *Porites*, *Acropora*, and *Platygyra* in tropical coral reefs (28). Thus, it has been speculated that the health and adaptability of coral holobionts are affected by the fungal community and its parasitism or infection. A prime example was that of *Aspergillus* leading to *Aspergillus aspergillosis* disease and high mortality of sea fans in West India, which satisfied Koch’s rule (46). Nevertheless, the link between coral-associated fungi and interspecific differences in coral heat tolerance remains unclear, and there is little information on the potential interactions between coral-associated fungi and other microbes (47, 48), especially the endosymbiotic Symbiodiniaceae, which is crucial for assessing the adaptability of coral holobionts to global warming and intensifying marine heatwaves.

**Fig 1:**
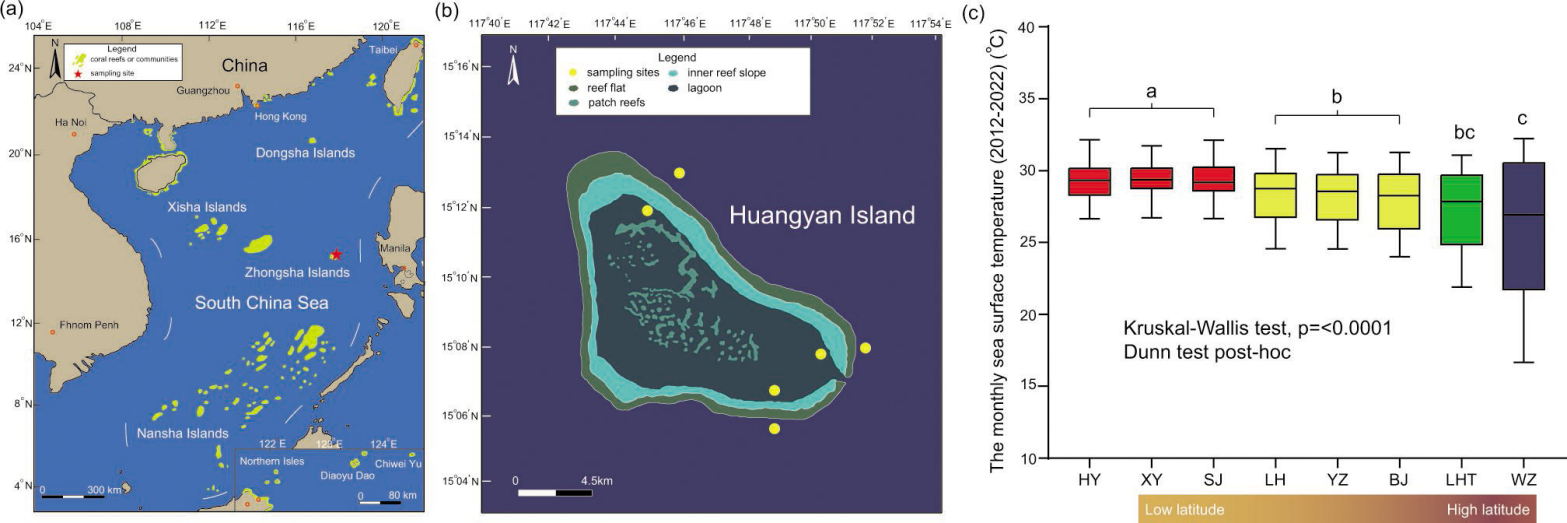
Study area and sampling sites. (a) Distribution of coral reefs or communities in the SCS. The red star denotes HYI; (b) physiognomy of HYI, an isolated atoll in the eastern SCS. The yellow points represent sampling sites; (c) monthly sea surface temperatures (2012–2022). Xinyi Reef (XY; 9°20′−9°21′ N, 115°54′−115°58′ E) and Sanjiao Reef (SJ; 10°10′−10°13′ N, 115°16′−115°19′ E) are located in the Nansha Islands in the low-latitude region of the SCS. Langhua Reef (LH; 16°0′−16°5′ N, 112°26′−112°35′ E), Yuzhuo Reef (YZ; 16°18′−16°21′ N, 111°57′−112°5′ E), and Beijiao (BJ; 17°06′−17°07′ N, 111°28′−111°31′ E) are distributed in the Xisha Islands in the intermediate-latitude region of the SCS. The fringe reefs of Luhuitou (LHT; 18°12′−18°13′ N, 109°28′−109°29′ E) and Weizhou Island (WZ; 21°00′−21°04′ N, 109°04′−109°08′ E) are located in the biogeographical transition zone and subtropical climate zone, respectively, both belonging to the northern part of the SCS. Different colors indicate the results of the Dunn test post-hoc analysis.

Huangyan Island (HYI; 15°13′48″–15°05′24″ N, 117°40′12″–117°52′00″ E; Fig. 1a and b) is located in the eastern section of the SCS. It is an isolated atoll and one of the major components of the Zhongsha Islands. A recent study found that the highest probability of coral thermal bleaching occurred at 15–20 latitudes north and south of the Equator, based on synthesizing coral bleaching events at 3,351 sites from 81 countries (1998–2017) (49). Thus, the coral reefs of HYI will probably experience enhanced thermal bleaching and stress in the future. The result of monthly average sea surface temperature (SST; 2012–2022) analysis showed that the SST of HYI (29.3°C ± 1.3°C) was higher than those of other coral reefs at the same latitude (e.g., Xisha Island; 28.1°C ± 1.9°C), which was similar to those of coral reefs at low-latitude regions (e.g., Nansha Islands; 29.4°C ± 1.1°C; Fig. 1c). The average Symbiodiniaceae density of corals (3.12 ± 0.11 × 10^6^ cells·cm^−2^) (50, 51), coral recruitment (15.7 ± 2.2 ind·m^−2^), and crustose coralline algae cover (42%) (52) in HYI was higher than those in other tropical coral reefs in the SCS. In addition, HYI is a submerged atoll and has strong water exchange between the outer reef slope and the lagoon (51, 53), which contributes to the overall environmental stability of HYI among the geomorphological belts. Thus, coral reefs of HYI have a thermal environment and robust ecological status, which provide a natural laboratory for exploring the effects of microbiome dynamics and interactions between Symbiodiniaceae and fungi on the heat tolerance of coral holobionts in intermediate-latitude regions with high coral thermal bleaching risk.

This study aimed to analyze the diversity and community structure of Symbiodiniaceae and fungi among 18 coral species in HYI and to evaluate the interspecific differences in microbial community flexibility. We constructed links between ecological indices (α-diversity and potential fungal pathogens abundance) of these microbial communities and coral thermal bleaching percentage in the 15–20°N regions of the SCS during the 2020 coral bleaching event (Table 1), which will assist us in explaining how distinct community dynamics of Symbiodiniaceae and fungal and pathogen abundance are linked to thermal susceptibility. Indicators of fungi and Symbiodiniaceae were also characterized in this study. Moreover, the molecular ecological network was used to explore potential interactions between dominant Symbiodiniaceae and fungi, which assisted in identifying key microbial drivers and establishing associations between the topological properties of the Symbiodiniaceae–fungi interaction network (SFIN) and coral thermal bleaching susceptibility. The results of this study will expand our knowledge of the effects of microbiome dynamics and interactions between Symbiodiniaceae and fungi on interspecific differences in coral heat tolerance in the context of global warming.

**TABLE 1:** The coral sample information in HYI and the thermal bleaching percentage of coral species in the 15–20°N regions of the SCS during the coral bleaching event of 2020

Coral family	Genus	Species	Number of samples	Thermal bleaching percentage
Merulinidae	*Goniastrea*	*Goniastrea retiformis*	3	94.12%
Acroporidae	*Acropora*	*Acropora nana*	4	87.50%
Merulinidae	*Favites*	*Favites halicora*	6	72.73%
Acroporidae	*Acropora*	*Acropora anthocercis*	5	70.00%
Acroporidae	*Isopora*	*Isopora palifera*	6	65.00%
Acroporidae	*Isopora*	*Isopora cuneata*	3	65.00%
Merulinidae	*Goniastrea*	*Goniastrea pectinata*	5	61.54%
Acroporidae	*Acropora*	*Acropora gemmifera*	5	60.61%
Merulinidae	*Coelastrea*	*Coelastrea aspera*	3	60.00%
Agariciidae	*Leptoria*	*Leptoria phrygia*	6	59.09%
Poritidae	*Porites*	*Porites lutea*	6	54.73%
Merulinidae	*Merulina*	*Merulina ampliata*	6	50.00%
Pocilloporidae	*Pocillopora*	*Pocillopora woodjonesi*	3	47.62%
Plesiastreidae	*Plesiastrea*	*Plesiastrea versipora*	4	43.75%
Merulinidae	*Dipsastraea*	*Dipsastraea speciosa*	3	33.33%
Pocilloporidae	*Pocillopora*	*Pocillopora verrucosa*	5	17.84%
Merulinidae	*Hydnophora*	*Hydnophora exesa*	5	5.00%
Fungiidae	*Fungia*	*Lobactis scutaria*	3	2.00%

## RESULTS

### The coral thermal bleaching percentage and environmental characteristics

Eighteen coral species exhibited varying degrees of thermal bleaching in the 15–20°N regions of the SCS during the coral bleaching event of 2020. The thermal bleaching prevalence has weak associations with coral skeletal morphology and polyps. *Goniastrea retiformis* populations experienced the most serious thermal bleaching, the average bleaching prevalence was 94.12% (Table 1). The *Acropora nana* (87.50%), *Favites halicora* (72.73%), *Acropora anthocercis* (70.00%), *Isopora palifera* (65.00%), *Isopora cuneata* (65.00%), *Goniastrea pectinata* (61.54%), *Acropora gemmifera* (60.61%), and *Coelastrea aspera* (60.00%) also showed severe thermal bleaching (Table 1). Additionally, *Leptoria phrygia* (59.09%), *Porites lutea* (54.73%), *Merulina ampliata* (50.00%), *Pocillopora woodjonesi* (47.62%), *Plesiastrea versipora* (43.75%), and *Dipsastraea speciosa* (33.33%) suffered intermediated thermal bleaching during the coral bleaching event of 2020. Nevertheless, *Pocillopora verrucosa* (17.84%), *Hydnophora exesa* (5.00%), and *Lobactis scutaria* (2.00%) exhibited lower thermal bleaching susceptibility than other coral species, with thermal bleaching percentages below 20% (Table 1).

**Fig 2:**
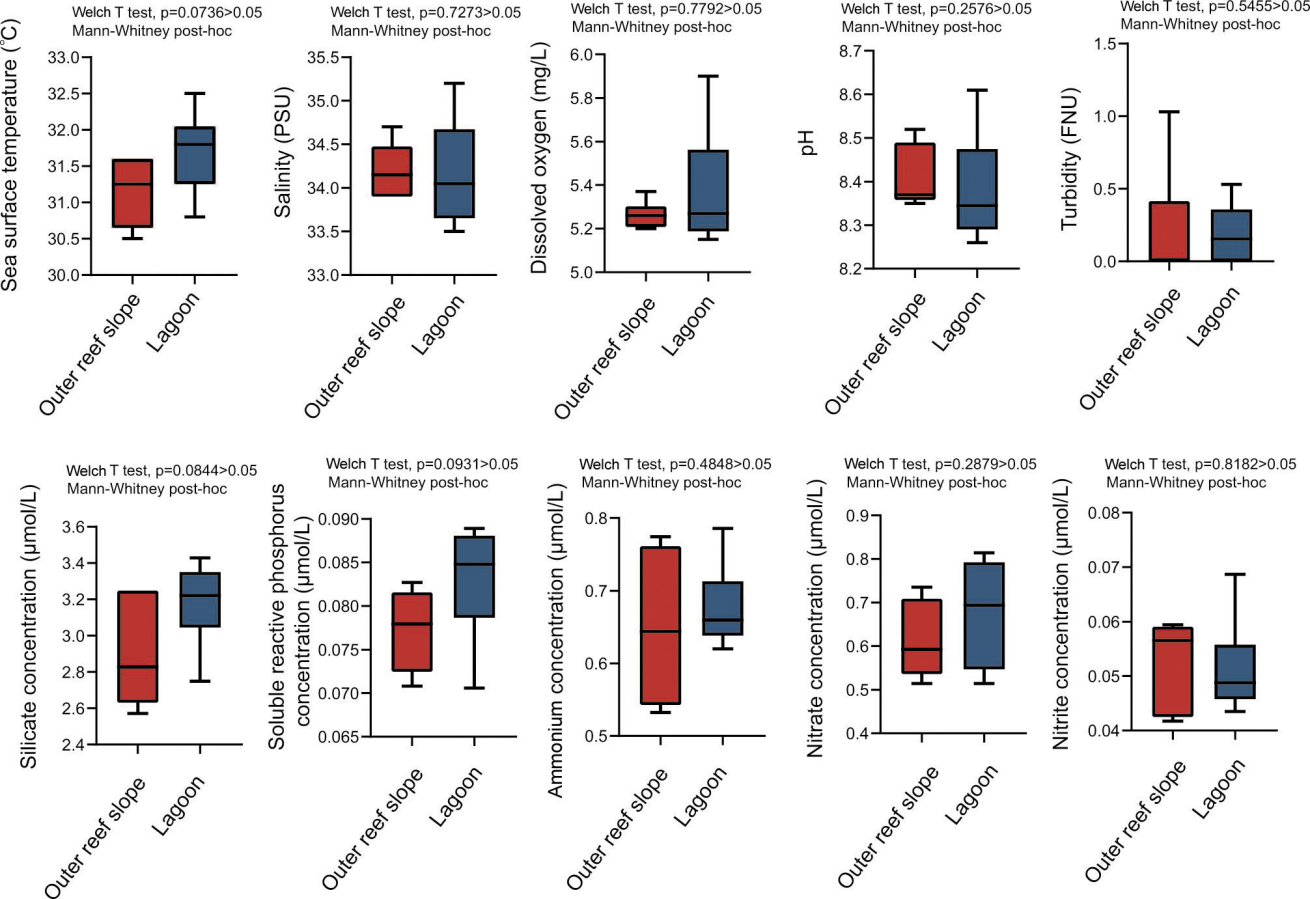
The statistical result of environmental factors between outer reef slope and lagoon in of coral reef in Huangyan Island.

Statistical analysis of the environmental parameters revealed that no significant differences were observed in the temperature (°C), salinity (PSU), DO (mg/L), pH, and turbidity (FNU) between the outer reef slope and the lagoon in HYI (Fig. 2; Table. S1). Although the concentration of SiO_3_^2−^, PO_4_^3−^, NH_4_^+^, and NO^3−^ of seawater in the lagoon was lower than that in the outer reef slope in HYI, there was also no significant difference of nutrients across the geomorphological belts. This phenomenon can be attributed to the close association of HYI with submerged atoll and strong water exchange.

### The diversity of Symbiodiniaceae and fungi

**Fig 3:**
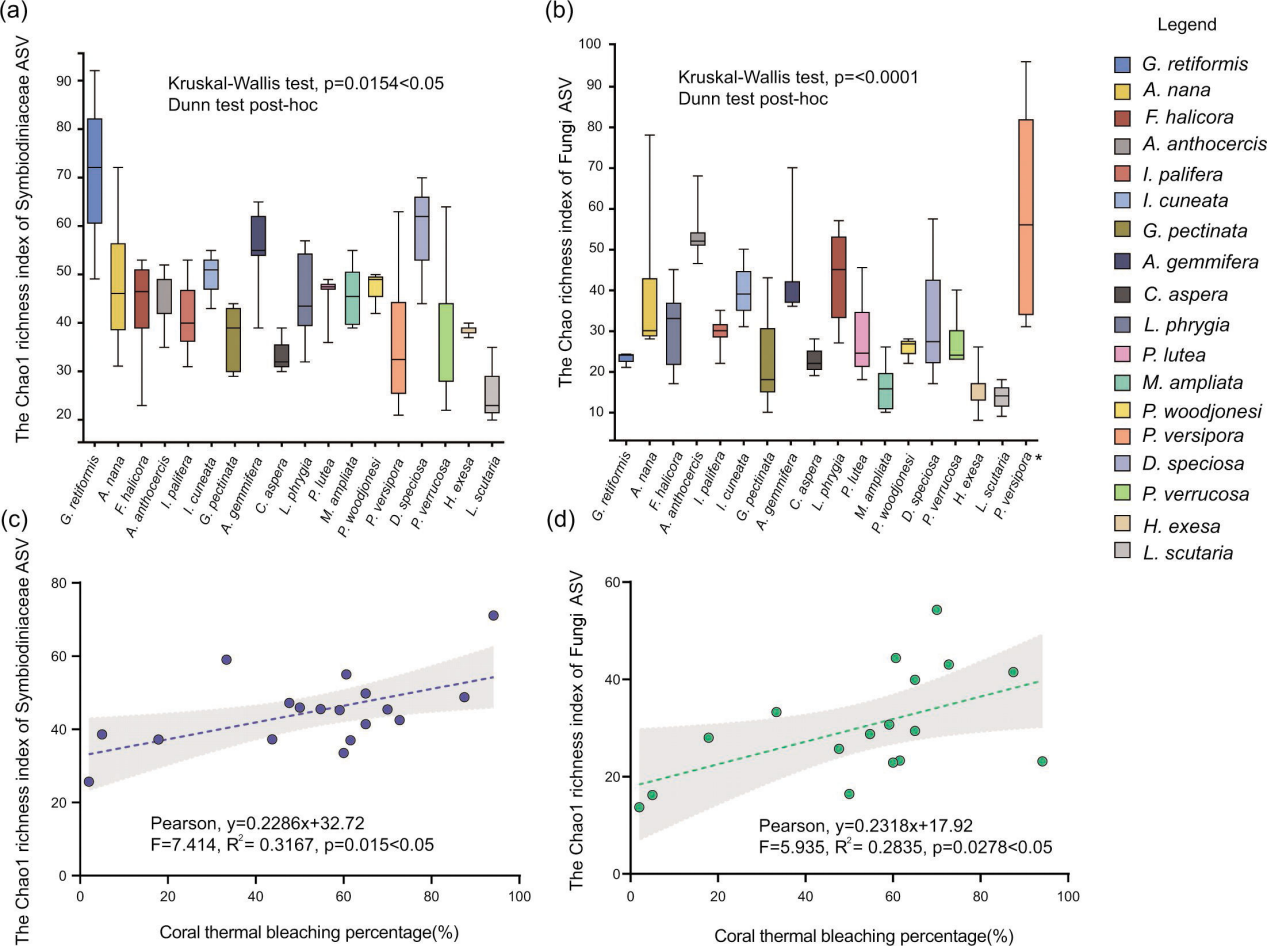
The α-diversity of Symbiodiniaceae and fungi of 18 coral species in HYI. The Chao1 richness index of (a) Symbiodiniaceae and (b) fungi of 18 species of corals in HYI; the correlation between the Chao1 richness index of (c) Symbiodiniaceae/ (d) fungi and the degree of coral susceptibility. The asterisk denotes abnormally high value of Chao1 richness index in *P. verispora*.

After blasting and filtering the reads, 5,251,966 Symbiodiniaceae ITS2 reads were obtained. *Cladocopium*, *Durusdinium*, and *Gerakladium* were identified in the 18 HYI coral species based on ITS2 sequence analysis after quality control (retaining ITS2 variants present with at least 1% abundance in at least one sample). At the sub-clade taxonomic level, 45 Symbiodiniaceae sub-clades were identified, of which the three genera were *Cladocopium* (*n* = 39), *Durusdinium* (*n* = 5), and *Gerakladium* (*n* = 1). In addition, 1,217 Symbiodiniaceae ASVs were identified after filtering and subsampling, which were used for Chao1 richness index statistics and to reduce the distraction of intragenomic variation. For the fungal community, the 2,142,129 sequences were aligned to fungi and clustered as ASVs after quality control and data set subsampling; there were 6 phyla, 26 classes, 55 orders, 119 families, 178 genera, 236 species, and 1,881 ASVs identified in 18 HYI coral species. In addition, there were significant differences in the Chao1 richness index of Symbiodiniaceae (Kruskal–Wallis test, *P* = 0.0154 < .05; Fig. 3a) and fungi (Kruskal–Wallis test, *P* < 0.0001; Fig. 3b) among HYI coral holobionts. The simple linear regression (SLR) analysis revealed that the coral thermal bleaching percentage was significantly and positively associated with the richness of Symbiodiniaceae (Pearson, *F* = 7.414, *R*^2^ = 0.3167, *P* = 0.015 < .05; Fig. 3c). Nevertheless, there was a significantly positive correlation between Chao1 richness index of fungi and thermal bleaching percentage of coral holobiont (Pearson, *F* = 5.935, *R*^2^ = 0.2835, *P* = 0.0278 < .05; Fig. 3d).

### The community structure of Symbiodiniaceae

**Fig 4:**
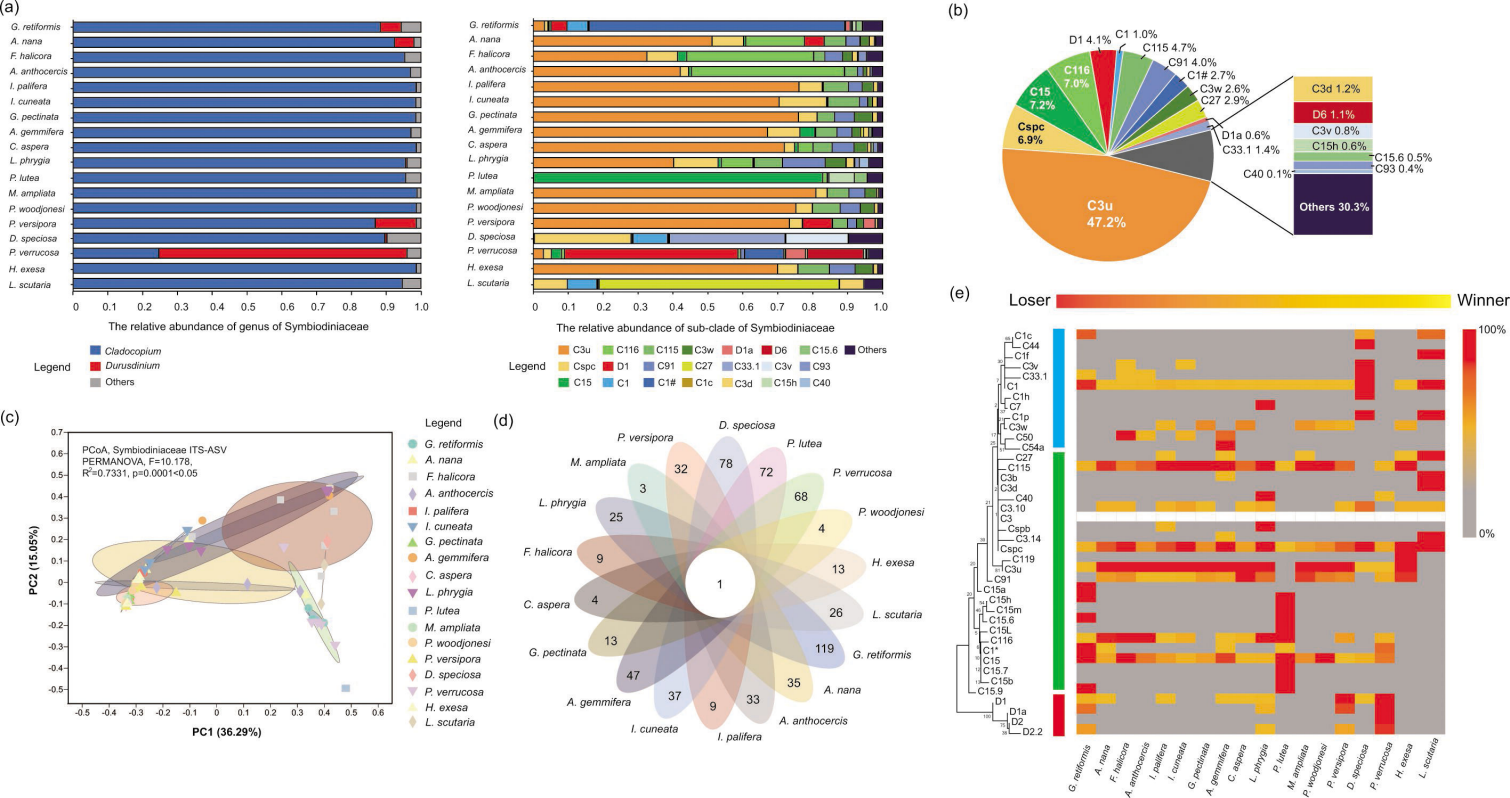
The community structure of Symbiodiniaceae among eighteen18 coral species in HYI in the SCS. (a) Relative abundance of genus and sub-clade of Symbiodiniaceae in 18 coral species in HYI; (b) community composition of Symbiodiniaceae of corals in HYI; (c) principal co-ordinates analysis (PCoA) of Bray–Curtis distances of Symbiodiniaceae ASV compositions associated with 18 coral species. Ellipses denote significant differences among 18 coral species [permutational multivariate analysis of variance (PERMANOVA)]; (d) the Venn diagram visualization for ubiquitous and specific Symbiodiniaceae ASV of 18 coral species; (e) abundance and enrichment characteristics of Symbiodiniaceae bio-indicator of 18 coral species in HYI based on ASV data set analyses. The blues and green rectangles denote thermally sensitive and tolerant Symbiodiniaceae sub-clades of *Cladocopium*, respectively, based on the results of phylogenetic analysis. The red rectangle represents heat-tolerant Symbiodiniaceae sub-clades of *Durusdinium*.

The Symbiodiniaceae community of coral in HYI was mainly dominated by *Cladocopium* (91.5% ± 17.1%) and *Durusdinium* (5.3% ± 16.8%). There were 21 dominant Symbiodiniaceae sub-clades (the relative abundance was >5% in at least one sample; Fig. 4a). The C3u sub-clade dominated the Symbiodiniaceae community of corals in HYI (42.7% ± 32.8%; Fig. 4b), which had a high relative abundance in 13 of the 18 coral species. However, the Symbiodiniaceae community of *G. retiformis* was dominated by C1# (70.3% ± 12.7%), C1 (8.2% ± 13.1%), and D1 (4.7% ± 6.0%), and the relative abundance of C3u was 3.2% ± 2.1%. In addition, *D. speciosa* had diverse dominant Symbiodiniaceae sub-clades: C33.1 (33.1% ± 1.0%), Cspc (27.8% ± 0.6%), C3v (17.8% ± 2.0%), and C1 (9.8% ± 1.0%) contributed high relative abundance to the symbiont community. It is worth noting that the relative abundance of *Durusdinium* was higher than that of *Cladocopium* in *Pocillopora verrucosa*, D1 (49.3% ± 18.0%) and D6 (15.6% ± 10.3%) were dominant in the Symbiodiniaceae community, but C1# (10.8% ± 18.1%) had the highest relative abundance in *Cladocopium*. Moreover, the C27 (68.8% ± 9.7%) and C15 (82.4% ± 5.5%) were predominant in the Symbiodiniaceae communities of *L. scutaria* and *P. lutea*, respectively.

The results of PCoA showed that there were significant differences in Symbiodiniaceae community structure among coral species in HYI (permutational multivariate analysis of variance; PERMANOVA, *F* = 10.178, *R*^2^ = 0.7331, *P* = 0.0001 < .05), and the 51.3% total variation in the Symbiodiniaceae community was explained by interspecific differences (Fig. 4c). The Venn diagram visualization revealed that the core symbiont microbiome had only one Symbiodiniaceae ASV (ASV 506), which aligned with the variant of the C3u sub-clade and lived in all HYI coral species (Fig. 4d). Moreover, the IndicSpecies test-based ASV data set analyses identified 45 indicators of the Symbiodiniaceae sub-clade among 18 coral species (Fig. 4e). The heat-sensitive C1 sub-clade and heat-tolerant Cspc, C3u, C91, C15, and C116 sub-clades were ubiquitous among coral species that have distinct thermal bleaching susceptibilities. It is worth noting that *Durusdinium* spp. do not show enrichment characteristics in all strong heat-tolerant coral species (e.g., *P. woodjonesi*, *H. exesa*, and *L. scutaria*) except *P. verrucosa*; these coral holobionts preferred to have symbioses with potential thermal-sensitive (e.g., C1 and *L. scutaria*) or tolerant *Cladocopium* (e.g., C115, C3u, Cspc, C119, C91, and *L. scutaria*; C115, C3u, and *P. woodjonesi*).

### The community structure of fungi

**Fig 5:**
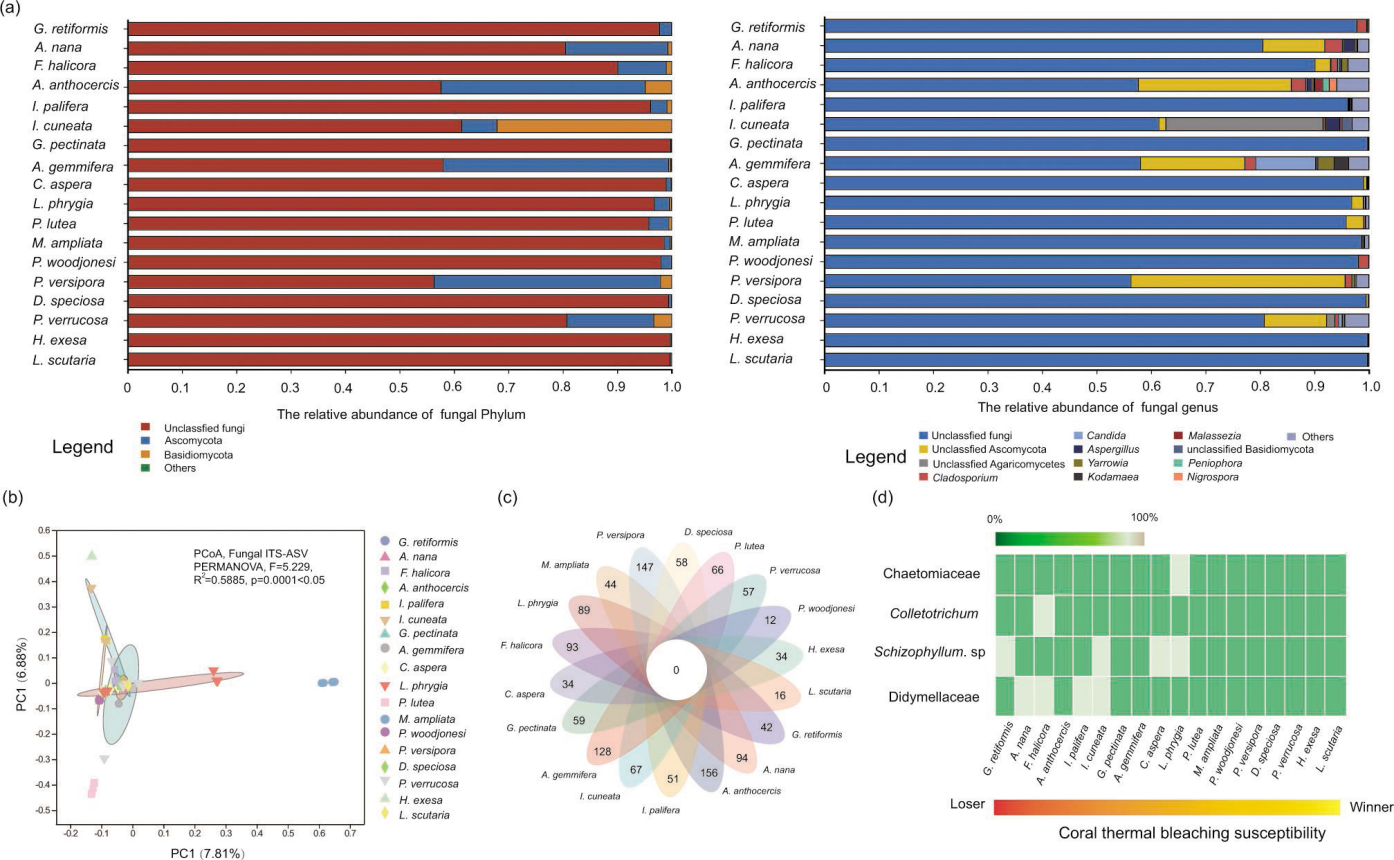
The community structure of fungi among 18 coral species in HYI in the SCS. (a) Fungal community composition for phylum, genus, and ASV levels of 18 coral species in HYI; (b) PCoA of Bray–Curtis distances of fungal compositions associated with 18 coral species. Ellipses denote significant differences among distinct coral species (PERMANOVA); (c) Venn diagram visualization for ubiquitous and specific fungal ASV of 18 coral species; (d) Abundance and enrichment characteristics of fungal bio-indicator of 18 coral species in HYI.

At the phylum level, the fungal community composition of coral holobionts in HYI was dominated by Ascomycota (10.4% ± 14.7%) and Basidiomycota (2.6% ± 7.5%), and a high abundance of unclassified fungi was identified in 18 coral species (87.0% ± 16.8%; Fig. 5a). Thus, corals in HYI have a stable community composition of fungi at the phylum level. In addition, the corals in HYI were mainly colonized by *Cladosporium* (0.9% ± 1.0%), *Candida* (0.7% ± 2.6%), *Aspergillus* (0.4% ± 0.7%), *Yarrowia* (0.4% ± 0.7%), *Kodamaea* (0.2% ± 0.6%), *Malassezia* (0.1% ± 0.4%), *Peniophora* (0.1% ± 0.3%), and *Nigrospora* (0.1% ± 0.3%) at the genus level, and the relative abundance of these fungal genera was >1% in at last one coral species. The mycobiome of coral also had abundant unclassified fungal taxa at the genus level, such as unclassified fungi (86.9% ± 16.8%), Ascomycota (6.7% ± 11.3%), Agaricomycetes (1.7% ± 6.8%), and Basidiomycota (0.1% ± 0.4%; Fig. 5a). However, the PCoA identified that the community structure of fungi was highly flexible among the distinct coral species (Fig. 5b). The results of the PERMANOVA test showed that there were significant differences in the fungal community structure among distinct coral species in HYI (PERMANOVA, *F* = 5.299, *R*^2^ = 0.5885, *P* = 0.0001 < .05). In addition, the Venn diagram visualization showed that there was no core fungal ASV present in all coral species and >80% of the samples. This suggests that the fungal community of corals in HYI has high variation and flexibility (Fig. 5c). Moreover, the IndicSpecies test found that Didymellaceae (indicator test, *P* = 0.043 < .05), *Schizophyllum* (indicator test, *P* = 0.035 < .05), *Colletotrichum* (indicator test, *P* = 0.040 < .05), and Chaetomiaceae (indicator test, *P* = 0.017 < .05) were indicators of distinct coral species in HYI. It is worth noting that Didymellaceae (0%–60.3%) and *Schizophyllum* (0%–47.1%) exhibited a higher relative abundance in coral species with high thermal bleaching susceptibility and *Colletotrichum* (relative abundance: >90%) and Chaetomiaceae (relative abundance: >90%) displayed relatively higher levels in *F. halicora* and *L. phrygia*, which demonstrate intermediate heat tolerance (Fig. 5d; Table.S2). However, the abundance of these four fungal indicators did not show any associations with coral species with stronger thermal adaptability.

### The functional traits of the fungal community

**Fig 6:**
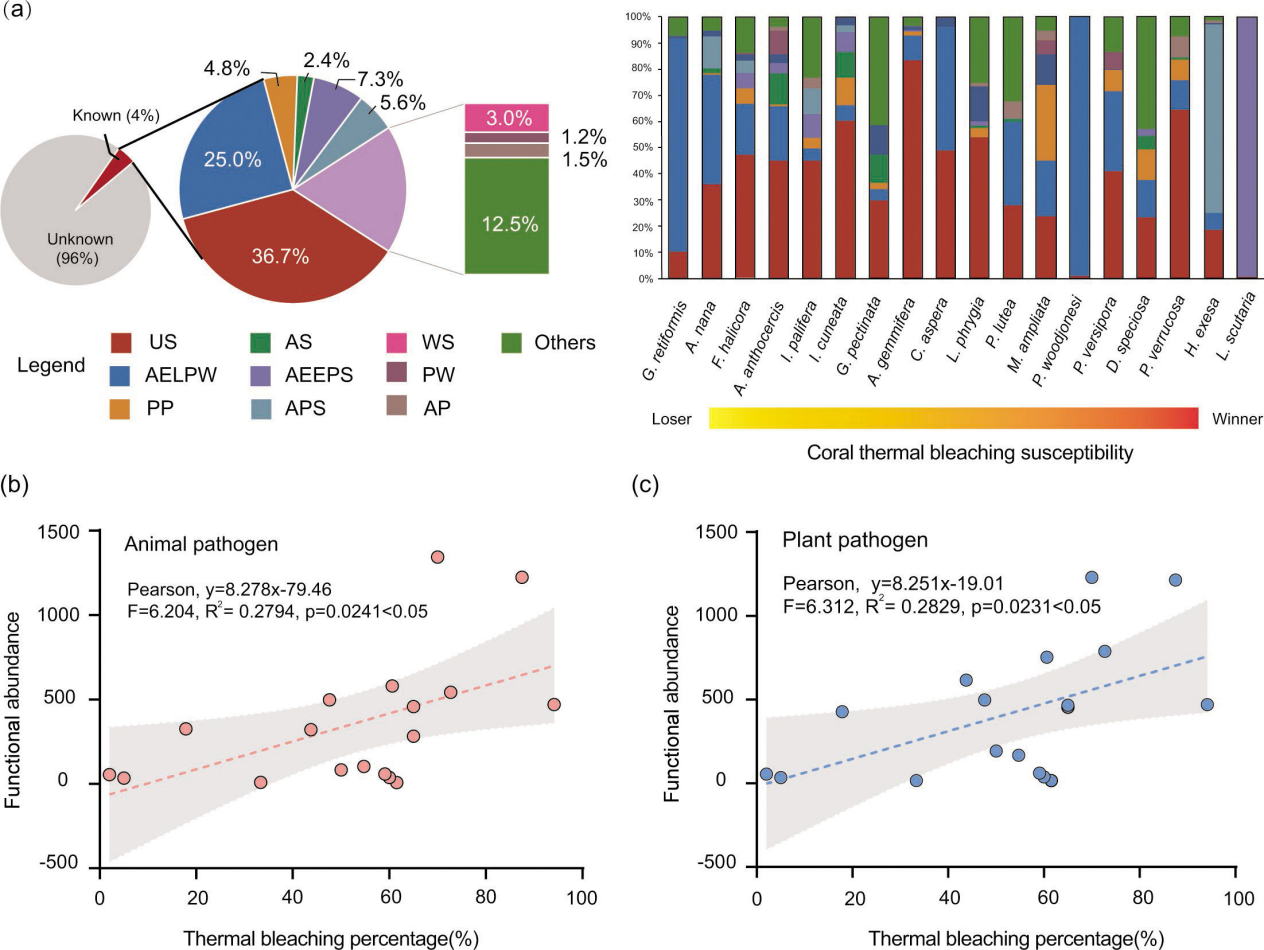
The relationship between the relative abundance of fungal pathogen function traits and coral thermal bleaching susceptibility. (a) Composition of fungi function group among coral species in HYI. The relative abundance of US, AELPW, PP, AS, AEEPS, APS, WS, PW, and AP was more than 1%; (b) There was a significantly positive correlation between the abundance of animal pathogen of fungi and coral thermal bleaching percentage (%). (c) There was a significantly positive association between the coral thermal bleaching percentage (%) and the abundance of fungal plant pathogen.

The results of the FUNGuild analysis showed that the ecological functions of 96% of fungi in the corals in HYI were unknown (Fig. 6a), which was consistent with the result of fungal community composition. The predictable ecological functional fungal community (4%) of corals in HYI was mainly colonized by undefined saprotrophs (US; 36.7% ± 22.1%) and animal pathogen–endophyte–lichen parasite–plant pathogen–wood saprotroph (AELPW; 25.1% ± 27.6%) fungi, which were distributed in almost all coral species, except for heat-tolerant *L. scutaria* and *H. exesa* (Fig. 6a). The known functional fungal community of *L. scutaria* was dominated by animal pathogen–endophyte–epiphyte–plant pathogen-undefined saprotroph (AEEPS; 99.4% ± 2.3%) fungi, and animal pathogen–plant pathogen-undefined saprotroph fungi (AEEPS; 72.1% ± 5.7%) had the highest relative abundance in the fungal community of *H. exesa* (Fig. 6a). In addition, the predictable functional fungal communities of HYI corals were also colonized by plant pathogen (PP; 4.8% ± 7.2%), animal pathogen-undefined saprotroph (AS; 2.4% ± 4.1%), animal pathogen-plant pathogen-undefined saprotroph (APS; 5.6% ± 17.0%), wood saprotroph (WS; 3.0% ± 4.4%), plant pathogen-wood saprotroph (PW; 1.2% ± 2.8%), and animal pathogen (AW; 1.5% ± 2.5%) fungi, and the relative abundance of these functional groups was >1% (Fig. 6a). Notably, fungal parasite-associated fungi were rare in the corals in HYI, and the total relative abundance of this functional fungal group was only 1.2%.

The results of SLR showed that the abundance of functional traits of animal pathogens in the fungal community had a significantly positive correlation with coral thermal bleaching percentage (Pearson, *F* = 6.204, *R*^2^ = 0.2794, *P* = 0.0241 < .05; Fig. 6b). In addition, the heat adaptability and acclimatization of coral holobionts also related with the reduction of plant-pathogenic fungi, because the thermal bleaching percentage of coral holobionts had a significantly positive association with the abundance of functional profiles of plant pathogens in the fungal community (Pearson, *F* = 6.312, *R*^2^ = 0.2829, *P* = 0.0231 < .05; Fig. 6c). Thus, an increase in the abundance of both animal- and plant-pathogenic fungi has closed associations with the increases of the thermal bleaching susceptibility of coral holobionts.

### The interaction and complexity of the microbiota of Symbiodiniaceae and fungi

**Fig 7:**
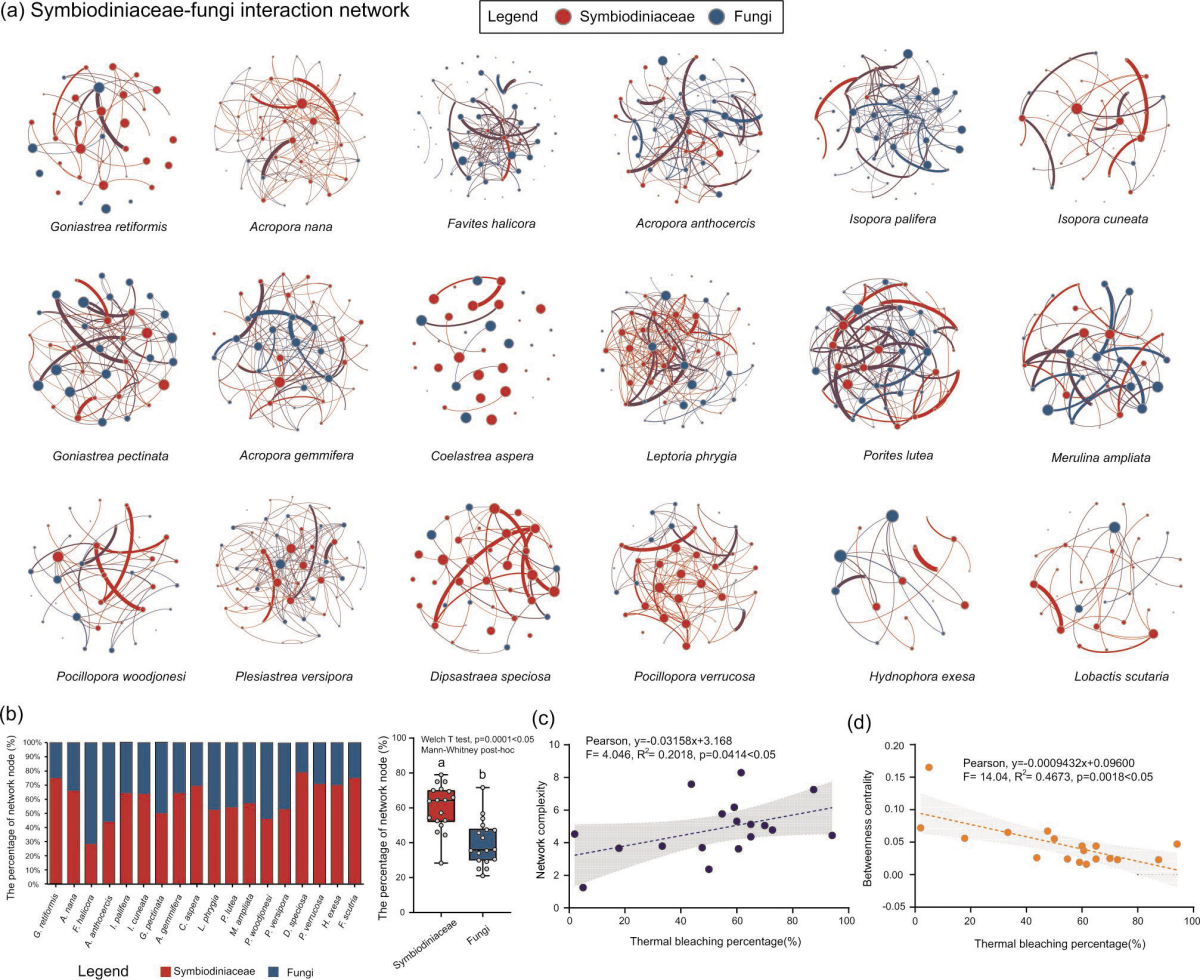
The correlation between the topological features of SFIN and coral bleaching susceptibility in HYI. (a) Molecular ecological interaction network of Symbiodiniaceae and fungi for 18 coral species in HYI, with co-occurrence relationships marked by edges of the network. Potential interactions of Symbiodiniaceae are denoted by red, the relationships among fungi in blue, and the co-occurrence association between Symbiodiniaceae and fungi in brown. (b) Percentage of network node for Symbiodiniaceae and fungi. (c) Correlation between complexity of SFIN and coral thermal bleaching percentage (%). (d) Relationship between betweenness centrality of SFIN and coral thermal bleaching percentage (%).

The results of molecular ecological network analysis (MENA) showed that there was a complexed potential interaction between dominant Symbiodiniaceae sub-clades (relative abundance > 5%) and fungi among distinct coral species (Fig. 7a). The average percentage of network node of Symbiodiniaceae was 60.2% ± 13.0% (ranging from 28.3% to 75.0%), which was significantly higher than those of fungi (39.8% ± 13.1%, ranging from 21.1% to 71.6%) in the SFIN of coral holobiont in HYI (Welch *t*-test, *P* = 0.0001 < .05; Fig. 7b). Symbiodiniaceae had the highest relative abundance of nodes in the SFIN of *G. retiformis*, whereas the highest percentage of fungal nodes was found in the SFIN of *F. halicora*. Therefore, the dominant Symbiodiniaceae sub-clades contributed more than fungi to the SFIN construction of the coral holobionts in HYI.

The SFIN patterns varied greatly among different coral species, as indicated by the multiple topological feature indices of the 18 networks. The total number of nodes ranged from 20 to 67, and the total number of links ranged from 25 to 320 (Table 2). In addition, there were differences in the average degree, network diameter, network centralization, average path length, clustering coefficient, and modularity of SFIN among the 18 coral species in HYI. The SFIN of *C. aspera* and *L. phrygia* had the weakest and strongest relationships among nodes, respectively. The average degree of SFIN of *C. aspera* was 0.144, whereas that of *L. phrygia* was 6.169 (Table 2), and the thermal bleaching susceptibilities of these two coral species were similar. In addition, coral species with distinct thermal bleaching tolerances had large scales of SFINs (ranging from 3 to 7; Table 2), and the SFIN diameter of *L. scutaria* with strong heat tolerance was equal to that of *A. gemmifera* with high thermal bleaching susceptibility. Regarding centralization and average path length, the SFIN of coral species with intermediate thermal bleaching susceptibility had the maximum and minimum of these topology parameters (e.g., *Coelastrea aspera*, *Favites halicora*, and *D. speciosa*). The modularity of the SFIN for coral species ranged from 0.342 to 0.800 in HYI, and the heat-sensitive *A. anthocercis* had the highest modularity (Table 2). Notably, SFIN complexity was significantly and positively correlated with coral thermal bleaching percentage (Pearson, *F* = 4.046, *R*^2^ = 0.2018, *P* = 0.0414 < .05; Fig. 7c). Nevertheless, there was a significantly negative association between the betweenness centrality of SFIN and coral thermal bleaching percentage (Pearson, *F* = 14.04, *R*^2^ = 0.4673, *P* = 0.0018 < .05; Fig. 7d). Thus, the coral thermal bleaching susceptibility is closely related to the complexity and betweenness centrality of SFIN.

**TABLE 2:** The topological features of Symbiodiniaceae–fungi interaction network for 18 species of coral in HYI

Species	Number of nodes	Number of edges	Average degree	Network diameter	Network centralization	Characteristic path length	Clustering coefficient	Modularity	Betweenness centrality	Complexity
*Goniastrea retiformis*	36	160	1.611	5	0.215	2.603	0.808	0.465	0.047	4.444
*Acropora nana*	44	319	4.818	4	0.207	1.971	0.741	0.586	0.023	7.250
*Favites halicora*	67	320	2.866	7	0.433	2.518	0.575	0.659	0.023	4.776
*Acropora anthocercis*	63	318	3.619	5	0.315	2.509	0.572	0.8	0.025	5.048
*Isopora palifera*	58	297	4.662	6	0.385	2.319	0.635	0.741	0.024	5.121
*Isopora cuneata*	36	157	1.778	7	0.316	2.487	0.783	0.73	0.044	4.361
*Goniastrea pectinata*	38	315	5.158	3	0.269	1.582	0.674	0.69	0.016	8.289
*Acropora gemmifera*	45	163	4.622	6	0.256	2.588	0.621	0.627	0.037	3.622
*Coelastrea aspera*	36	191	0.444	4	0.192	1.58	0.86	0.342	0.044	5.306
*Leptoria phrygia*	59	364	6.169	5	0.351	2.093	0.526	0.452	0.019	6.169
*Porites lutea*	46	265	5.87	6	0.453	2.064	0.559	0.684	0.024	5.761
*Merulina ampliata*	35	83	3.257	7	0.164	2.802	0.349	0.699	0.055	2.371
*Pocillopora woodjonesi*	39	144	1.897	3	0.15	1.662	0.835	0.666	0.067	3.692
*Plesiastrea versipora*	53	402	5.019	5	0.296	2.319	0.818	0.645	0.026	7.585
*Dipsastraea speciosa*	38	144	2.105	8	0.107	3.254	0.79	0.543	0.065	3.789
*Pocillopora verrucosa*	41	150	4.829	7	0.238	2.524	0.607	0.791	0.056	3.659
*Hydnophora exesa*	20	25	1.9	4	0.361	2.044	0.523	0.647	0.165	1.250
*Lobactis scutaria*	36	163	1.389	7	0.25	2.534	0.773	0.453	0.072	4.528

## DISCUSSION

### The heat-tolerant C3u sub-clade and *Durusdinium* may provide extra thermal acclimation to coral holobionts

The endosymbiotic Symbiodiniaceae communities of coral holobionts in the HYI are dominated by *Cladocopium* and *Durusdinium*. This is consistent with the symbiont composition of corals in the Indo-Pacific regions (29, 34, 35, 54, 55). Although HYI has an approximate latitude with the Xisha Islands in the intermediate latitudes of the SCS, the average monthly SST of HYI is not significantly different from those of low-latitude regions with serious heat stress (Fig. 1c) (35, 50, 51). It is worth noting that heat-tolerant C3u contributed the highest relative abundance to *Cladocopium* in the Symbiodiniaceae communities of HYI and was consistently sustained as a core symbiont in 18 coral species (Fig. 4a), suggesting that the high abundance and stable presence of C3u may associate with lower thermal bleaching susceptibility of coral holobiont in HYI. Previous studies have found that the Symbiodiniaceae communities of *Acropora*, *Fungia*, *Platygyra*, *Symphyllia*, *Favites*, *Goniastrea*, and *Pavona* were dominated by C3u and *D. trenchii* (D1–4 sub-clades) in hot coral reefs in the Andaman Sea in the northeastern Indian Ocean, which is a part of a massive warm water zone encompassing Southeast Asia, Indonesia, and northern Australia. The abundance of C3u was higher than that of *D. trenchii* in offshore reefs with increasing seawater transparency (31, 56). In addition, the C3u also contributed high relative abundance (29.4% ± 21.2%) in the Symbiodiniaceae community composition of corals in the southern SCS that was controlled by long-term thermal stress (35, 57, 58), and about 2/3 of coral species was colonized by C3u across outer reef slopes and lagoons in these regions (50, 59). Phylogenetic and ecological studies have found that the C3u sub-clade was derived from C3 after the Late Miocene or Early Pliocene based on ITS2 gene marker (Fig. 4e), making it a potential recent heat-tolerant ancestor of *Cladocopium* (35, 60). The relative abundance of C3u sub-clade in coral holobionts has significantly positive associations with SST and photosynthetically active radiation across 19 latitudes of the SCS (PAR) (35).

Interestingly, heat-tolerant C3u and *Durusdinium* (D1 and D6) have the ability to dominate the Symbiodiniaceae community of the same coral samples or species in HYI and other low-latitude coral reefs (Fig. 4a), for example, *Echinopora*, *Pocillopora*, and *Diploastrea* have symbioses with C3u and D1 in the Perhentian Islands and Redang Islands in Malaysia (61). This symbiotic characteristic has also been identified in corals from the Gulf of Thailand (e.g., *Platygyra daedalea*) and the Philippine Archipelago (62, 63), suggesting that corals prefer to establish symbioses with heat-tolerant C3u and *Durusdinium* at the same time in long-term and stable thermal stress environment. It has been generally recognized that *Durusdinium* is a heat-tolerant Symbiodiniaceae that can provide 1.0°C–2.0°C of additional thermal tolerance to the coral holobiont (3, 29, 56, 64). A recent study found that coral holobionts shifted the dominant symbiont of the Symbiodiniaceae community from *Cladocopium* to *Durusdinium* in the central equatorial Pacific Ocean during the 2015–2016 El Niño event. This enhanced the survival and resilience of corals during long-term marine heatwaves (6). However, calcification and photosynthetic efficiency in coral hosts harboring *Durusdinium* are greatly reduced compared with those of corals harboring native *Cladocopium* (56, 65). Thus, the coral has symbioses with heat-tolerant C3u and *Durusdinium*, which may assist the coral to improve heat resistance potential and avoid the negative impact of symbioses on the growth and health of the holobiont. It has been found that *P. verrucosa*, *G. retiformis*, and *A. nana* had higher live coral cover (9.16%, 3.69%, and 0.89%, respectively) and dominance (0.086, 0.032, and 0.032, respectively) than other coral species in thermal HYI (53, 66), which simultaneously established symbioses with heat-tolerant C3u and *Durusdinium*. Nevertheless, corals also have the ability to establish symbioses with diverse sub-clades of *Cladocopium* or have specific symbioses with only one *Cladocopium* taxon (Fig. 4a). This leads to significant interspecific differences in Symbiodiniaceae community structure among coral species in HYI. It was worth noting that these Symbiodiniaceae sub-clades were mostly derived from C3 (Cspc, C27, and C91) or C15 (C116 and C115) (31, 35, 60), which may have inherited thermal adaptability from a potential heat-tolerant ancestor. It has been widely reported that C27- *L. scutaria* and C15- *P. lutea* symbioses have a low thermal bleaching susceptibility (4, 9, 67–69). Accordingly, the Symbiodiniaceae community of coral in HYI was primarily characterized by the prevalence of heat-tolerant sub-clades of *Cladocopium* and *Durusdinium*, which may potentially equip the corals in HYI with increased thermal tolerance, enabling them to respond to heat stress condition akin to those experienced in low-latitude regions.

### The increase of fungal diversity and pathogen abundance was associated with higher thermal bleaching susceptibility of corals

This study found that the fungal alpha diversity and the richness of unique fungal ASVs have a significantly positive correlation with thermal bleaching percentage of corals in HYI, which suggests that an increase in fungal diversity and richness was associated with the higher coral thermal bleaching susceptibility. Fungal diversity is closely associated with environmental stress levels, such as SST, depth, nutrient concentration, and disease (39, 70, 71). For example, the fungal diversity of *Acropora loripes* increased with water depth and available nutrients in the Gulf of Aqaba, and the fungal diversity of lesioned coral colonies was higher than that of healthy coral colonies (45). In addition, Amend et al. (44) showed that the fungal community of *Acropora hyacinthus* in warmer habitats contained more phylogenetic diversity than that of *A. hyacinthus* in colder habitats of Ofu Island in American Samoa (44). Notably, metagenomic analyses have revealed fungal proliferation and increased zoosporic members under thermal or environmental stress (71–73). Thus, this study suggests that the diversity and abundance of potentially opportunistic or pathogenic fungi have closed associations with interspecific difference of thermal bleaching susceptibility in coral. Additionally, the activity of pathogenic fungi has a negative ecological impact on corals, Symbiodiniaceae, and endolithic algae in heat-stressed environments. The results of the FUNGuild analysis showed that an increase in the abundance of animal and plant pathogenic fungi was related with higher coral thermal bleaching susceptibility, and heat-tolerant coral species (e.g., *L. scutaria*, *H. exesa*, and *P. woodjonesi*) have a low functional abundance and diversity of pathogenic fungi (Fig. 6b and c). Nevertheless, a substantial proportion of fungi taxa in corals of HYI remained unclassified (87.0% ± 16.8%), and only 4% of fungal ecological functions has been identified. Hence, the potentially positive or negative impact of these unidentified mycobiome on heath state and thermal tolerance susceptibility of coral holobiont need to be further studied.

Notably, there were no core fungal ASVs among the 18 coral species in HYI, suggesting that the fungal communities of corals have high flexibility and interspecific heterogeneity. Coral holobionts share the same fungal taxa at phylum and class levels globally, for example, ascomycetes have been identified in almost all ecological and microbiological studies on coral–associated fungi (28, 39, 43, 45, 73–75), and Sordariomycetes, Dothideomycetes, Eurotiomycetes, and Saccharomycetes are widely distributed across coral reefs worldwide (42, 71, 76). However, the core fungal taxa were rare at the species, operational taxonomic units (OTUs), and ASV levels, even for one coral species in the same region. Only 11 core fungal OTUs from four classes have been identified in 90% of *A. hyacinthus* colonies (*n* = 36) based on 454 DNA amplicon sequences in the Red Sea (44). The numbers of core fungal ASVs or OTUs of corals were much less than those of bacteria and Symbiodiniaceae (Fig. 5d) (77–79), which suggests that phylogenetic association and coevolution between corals and fungi was limited, and the flexibility of the fungal community might be higher than that of the bacterial community in coral holobionts. However, the intragenomic polymorphism of eukaryotic cell may also play a role in the variability and flexibility of fungal community. Interestingly, changes in fungal diversity were similar to those of bacterial diversity; they all had positive associations with global or local environmental stress level of coral. Thermal or other stress factors (e.g., low pH, human interference, and algal contact) tend to increase bacterial alpha diversity (80–85), because the coral holobiont is an open microbial system (17, 86), and microbial invasion and heat stress disrupt the microbiome function and increase the number of microbes not typically resident in corals (85, 87). Additionally, it has been found that the fungi skewed toward having a negative impact on the heath state of coral holobionts under the environment stress influence (39), and the invasive, parasitic, opportunistic, or pathogenic fungi had close associations with coral disease (e.g., dark spot syndrome and aspergillosis) (88, 89). Thus, corals with high susceptibility to thermal bleaching may have weaker resistance to the increase and invasion of opportunistic or pathogenic fungi in heat-stressed environments. Moreover, there were significant differences in the fungal community structure among distinct coral species in HYI (Fig. 4b); however, there was no significant association between fungal community dissimilarity and coral thermal bleaching susceptibility (Pearson, *R*^2^ = 0.0014, *P* = 0.735 > .05; Fig. S1a). Thus, changes in the beta diversity of fungal communities differ from those of bacteria (23, 82, 86, 90). However, fungal alpha diversity has a negative association with fungal beta diversity within the coral holobiont (Pearson, *R*^2^ = 0.0489, *P* = 0.047 < .05; Fig. S1b), which was mainly contributed by pathogenic fungi and may have been closely associated with the process of fungal mycoparasitism or hyperparasitism (the functional profile abundance of fungal pathogen was 1.2% in HYI; Fig. 6a) (91).

### The fungal indicators of coral thermal bleaching susceptibility and their potentially ecological function

The four fungal indictors Didymellaceae, *Schizophyllum*, *Colletotrichum*, and Chaetomiaceae were identified in corals of HYI in this study and were used to classify the interspecific differences in the fungal community and thermal bleaching susceptibility (Fig. 5e). Although these fungal indicators have rarely been explored or researched in marine ecosystems (91), it is interesting that the vast majority of fungi sampled from marine surfaces or deep-sea environments branch close to or within clades of known terrestrial fungi, suggesting that some core or key ecological function traits may be shared among fungi with adjacent divergence, and many fungi have the ability to easily transition to distinct marine ecosystems (39, 92). The ecological function of fungal indicators in coral holobionts has been speculated by comparing them with fungal communities in distinct creatures or ecosystems. Notably, the fungal indicator Didymellaceae showed high relative abundance in heat-sensitive coral species; this may be closely associated with coral thermal bleaching. Didymellaceae are able to live in the ocean (93) and have also been isolated from sponges (e.g., *Callyspongia* sp.) in coral reefs (94). Many primary plant pathogens have been found in Didymellaceae, such as *Phoma*, *Ascochy*, and *Didymella*, which can lead to serious diseases in Cruciferae and oilseed rape (95, 96). Thus, an increase in the abundance of Didymellaceae may be associated with a reduced stability of coral–Symbiodiniaceae symbioses under thermal stress. Interestingly, *Colletotrichum* was enriched in the fungal community of coral species with intermediate thermal bleaching susceptibility levels (e.g., *F. halicora*), which may be a specific opportunistic plant pathogen that mediates coral bleaching by parasitizing Symbiodiniaceae under heat stress. *Colletotrichum* is recognized as one of the top 10 fungal plant pathogens that can cause anthracnose spots and blights in aerial plants and food crops (97–100). It is worth noting that the *Colletotrichum* has a unique intracellular hemibiotrophic lifestyle, enabling it to establish infection through a brief biotrophic phase, and some species can live in subcuticular tissues (98). This physiological characteristic may provide a basis for the invasion of symbiosomal cells (Symbiodiniaceae microhabitats) into coral gastrodermis (17). Owing to latent infections (101), the destructive and necrotrophic phases of *Colletotrichum* may be activated by heat stress. This induces the necrosis of Symbiodiniaceae cells by producing narrower secondary hyphae, which may lead to coral thermal bleaching (102, 103). This study also found a complex interaction between fungi and Symbiodiniaceae and that the complexity of the SFIN had a significantly negative association with coral thermal tolerance (Fig. 7c).

However, Chaetomiaceae may be beneficial for coral holobionts, which have been identified in aquatic ecosystems and are closely associated with marine invertebrates (e.g., *Cladiella* sp. and *Apostichopus japonicas*) (104–106). Many members of Chaetomiaceae can produce chaetoglobosins, which have strong antibacterial activity (107), and diverse chaetoglobosins have been isolated from *Pocillopora damicornis*-associated Chaetomiaceae (e.g., *Chaetomium globosum* C2F17) in the SCS (106). It has been found that the pathogenic bacteria have the ability to mediate coral thermal bleaching. For example, pathogenic *Vibrio* (*Vibrio shiloi*, *Vibrio coralliilyticus*, and *Vibrio* AK-1), Acidobacteria, and Flavobacteriales are closely associated with coral thermal bleaching (23, 86, 108, 109), and *Escherichia coli* is sustained in the core bacterial microbiota of *P. verrucosa* in thermal tropical coral reefs (85). Thus, heat-sensitive coral species may acclimate to thermal stress by enriching beneficial Chaetomiaceae species with antimicrobial activity against coral pathogens. In addition, it was verified that the fermentation broth extracts of Chaetomiaceae have antioxidant activity and that the antioxidant capacity of the extracts was comparable to that of Vitamin C (110), which may assist coral holobionts in responding to oxidative stress induced by high temperatures. Transcriptomic studies have found that heat stress leads to strong expression of photoprotective coral host pigments (20), and genes involved in cellular and oxidative stress responses of coral hosts are upregulated under heat stress conditions (21, 111–113). Thus, coral species with an intermediate thermal bleaching susceptibility (e.g., *L. phrygia*) may respond to seawater warming by increasing the abundance of Chaetomiaceae. The fungal indicator *Schizophyllum* might be a beneficial fungus, which is distributed in coral species with intermediate and high susceptibility to thermal bleaching. Furthermore, marine *Schizophyllum* may be derived from terrestrial ecosystems and plays an important role in carbon cycling in the ocean (114). Moreover, it was recently isolated from corals in the tropical coral reefs surrounding Hainan Island (115). Notably, marine *Schizophyllum* has strong anti-*Vibrio* activities, and some nontoxic strains of *Schizophyllum* (e.g., *Schizophyllum commune* MCCCZ16) have efficacy against *vibrio vulnificus* during infection of white shrimp (116). Therefore, *Schizophyllum* may assist heat-sensitive coral holobionts in resisting thermal bleaching mediated by *Vibrio* (23, 109).

Accordingly, Didymellaceae, *Schizophyllum*, *Colletotrichum*, and Chaetomiaceae are potential fungal indicators of coral thermal bleaching susceptibility levels. Pathogenic or opportunistic fungal indicators may be associated with a decrease of the heat tolerance of coral–Symbiodiniaceae symbioses. However, heat-sensitive coral species may enrich potentially beneficial fungi (Chaetomiaceae and *Schizophyllum*) to respond to heat stress and pathogen activity.

### The interaction of Symbiodiniaceae and fungi will affect the thermal adaptive potential of coral holobionts

There was a complex interaction between Symbiodiniaceae and fungi in the coral holobionts of HYI (Fig. 7a). Although, the interaction direction between Symbiodiniaceae and fungi in SFIN was not clear, the number of Symbiodiniaceae nodes was significantly higher than that of the fungal nodes in the SFIN (Fig. 7b), implying that the dominant Symbiodiniaceae sub-clade (relative abundance >5%) was the driver in the SFIN. Some dominant members of *Durusdinium* have been found to improve environmental adaptability by inhibiting parasitic symbionts (e.g., *Durusdinium trenchii* and C7) (117, 118). In addition, MENA studies found that dominant Symbiodiniaceae establish many co-occurrence relationships with rare symbiont biospheres and can regulate the Symbiodiniaceae–bacteria interaction network (SBIN) in coral holobionts (37, 38, 85, 117). For instance, *Cladocopium* dominated in the Symbiodiniaceae communities of endemic coral species in tropical and subtropical coral reefs in the SCS (35, 69, 85, 119), which was a driver of SBIN and controlled and regulated microbial networks by cooperating with α- and γ-proteobacteria (85). In addition, coral Symbiodiniaceae communities are assembled by 2–5 to dominant and diverse rare symbionts (64, 120, 121), and dominant Symbiodiniaceae establish the most stable symbioses with coral hosts owing to long-term acclimatization and coevolution (28, 122, 123). These symbioses were found to be optimal for coral growth and development (3), and the dominant Symbiodiniaceae taxa may maintain or improve the health of corals by regulating the SFIN. Topological feature analysis showed that the diameter, centralization, average degree, average path length, and clustering coefficient of the SFIN were characterized by interspecific heterogeneity (Table 2), suggesting that the heat tolerance of the coral holobiont was not affected by the scale, structural robustness, efficiency of information, or energy transport of SFINs (124–127). Moreover, niche specialization of the microbial community of coral holobionts can be reflected by modularity (127, 128), but there was a weak association between the modularity of SFIN and coral heat tolerance in this study. Although heat-sensitive *A. anthocercis* had the highest modularity, which may imply a high degree of niche differentiation of the microbial community constructed by Symbiodiniaceae, fungi might reduce the thermal adaptive potential of coral holobionts, niche specialization of the microbial community, and other topological properties of SFINs, which were mainly shaped by interspecific differences among coral species.

It is worth noting that the complexity and betweenness centrality of the SFIN had negative and positive correlations with coral thermal bleaching tolerance, respectively (Fig. 7c and d). These results indicate that the coexistence pattern and interaction of Symbiodiniaceae and fungi affect the heat tolerance of coral holobionts, and that the decrease in complexity and increase in the betweenness centrality of SFIN may improve the heat tolerance of coral holobionts. In previous studies, the high complexity of microbial networks indicated stronger stability and stress resistance of microbial communities in terrestrial ecosystems and animal guts (127, 129–131). However, coral holobionts that survive from stressful environments tend to have lower microbial interaction complexity (85, 132). Healthy coral holobionts showed low microbial network complexity under organic pollution, high temperature, low salinity, and acidification conditions, which have been reported in coral reefs in the SCS and western Atlantic at distinct spatial scales (e.g., latitudinal gradient, upwelling zone, and nitrogen content gradient) (85, 133, 134). Interestingly, a large-scale investigation of the coral microbiome showed that an increase in microbial network complexity may reduce the environmental adaptability of coral holobionts, because pathogenic or opportunistic microorganisms are drivers of these increase mechanisms. This indicates the destabilized microbiome and dysbiosis of coral (23, 82). Although these conclusions were suggested by the coral-associated bacterial community, the change in the alpha diversity of fungi was similar to that of bacteria in coral holobionts (28, 44, 86, 87), and the richness index of the fungal community was negatively correlated with coral heat tolerance (Fig. 3b). Therefore, the decrease in network complexity of the SFIN may be attributed to the decline in the parasitism activity of fungi on Symbiodiniaceae. This is closely associated with the regulation and immunity of coral–Symbiodiniaceae symbioses and increases the heat tolerance of coral holobionts (23). In addition, coral-associated microbial interaction networks characterized by high values of betweenness centrality may have stronger resilience because the removal of nodes does not greatly shape the connectivity of others (132, 135). *Pseudodiploria strigose* colonies were able to acclimate to temperature fluctuation in the inner reefs of Bermuda (annual temperature ranged from 13°C to 15°C), and the microbial network betweenness centrality of the surface mucus layer of this species in the inner reef was higher than that in the outer reefs (annual temperature fluctuation was 10°C) (132). Thus, an increase in betweenness centrality potentially confers more resilience to the microbial community composed of Symbiodiniaceae and fungi, which improves the adaptive potential of coral holobionts to heat stress.

Accordingly, the dominant Symbiodiniaceae taxa were drivers of the SFIN, which may affect the health of coral holobionts by regulating the coexistence pattern between Symbiodiniaceae and fungi. The thermal bleaching susceptibility of coral holobionts is closely associated with potential interactions between Symbiodiniaceae and fungi, and the low complexity and high resilience of SFINs may contribute to the stronger heat tolerance of coral holobionts.

### Conclusion

This study found that the Symbiodiniaceae community of corals in HYI was dominated by heat-tolerant *Cladocopium* (C3u sub-clade) and *Durusdinium* (D1 and D6 sub-clades), which may provide extra heat-adaptive potential to coral holobionts and assist corals to acclimate to long-term thermal stress. There were no core fungal ASVs in the coral holobiont, suggesting that the fungal community had high interspecific heterogeneity and flexibility. However, fungal diversity and the abundance of pathogens have significantly positive correlations with coral thermal bleaching percentage. Thus, the increase in fungal diversity and pathogen abundance was closely linked to higher thermal bleaching susceptibility in coral holobionts. Notably, there were four distinctive fungal indicators associated with coral species in HYI. These indicators consist of potentially pathogenic Didymellaceae and Chaetomiaceae, as well as speculatively beneficial *Schizophyllum* and *Colletotrichum*, although their function requires further validation. These four fungal indicators exhibit associations with thermal bleaching susceptibility of coral holobionts, varying from high to intermediate levels. Moreover, there were complex interactions between Symbiodiniaceae and fungi in coral holobionts, and the dominant Symbiodiniaceae was the main constructor and driver of SFINs. This may affect the coral heath state by regulating the coexistent pattern of Symbiodiniaceae and fungi. Coral thermal bleaching susceptibility is closely associated with the topological properties of the SFIN. Low complexity and high betweenness centrality may indicate limitations in fungal parasitism activity and strong microbial network resilience, which will improve the heat tolerance of coral holobionts. Our study highlights the ecological effects of microbiome dynamics and interactions between Symbiodiniaceae and fungi on coral thermal bleaching susceptibility, providing insights into the role of microorganisms and their interaction as drivers of interspecific differences in coral thermal bleaching.

## MATERIALS AND METHODS

### Coral thermal bleaching percentage and environmental parameter measurements

The information of coral thermal bleaching percentage was obtained and reanalyzed from coral reef survey in the 15–20°N regions (Hainan Island, Xisha Island, and Zhongsha Islands) of SCS in the coral thermal bleaching event of 2020 (136–138). The data set of coral reef surveys of 2020 was constructed by line intercept transect techniques (atoll: 5–15 m; fringing reef: 2–6 m) and Point Intercept Transect video re-sampling (66, 139, 140) according to our benthic surveys and previous studies (137, 140, 141). Coral species were identified following taxonomic criteria (142, 143). The three levels were used to score the degree of thermal bleaching severity: Level 1, minor thermal bleaching and healthy (0%–20% bleached); Level 2, medium thermal bleaching (20%–60% bleached); and Level 3, severe thermal bleaching and dead (60%–100% bleached) (144). The percentage of coral thermal bleaching was determined as the thermal bleaching cover (all three level) of coral species relative to the total cover of this coral species, which can indicate coral thermal bleaching susceptibility (Table 1).

A total of 30 seawater samples (5 L/sample) were collected in six study sites from the outer reef slope and lagoon in HYI. The temperature (°C), salinity (PSU), DO (mg/L), pH, and turbidity (FNU) were measured by ProDSS Multiparameter Digital Water Quality Mater (YSI, USA) at the same time as that of water sample collection. Consequently, the seawater was stored at −80°C for nutrient tests, and NO^3−^ (μmol/L), NO_2_^−^ (μmol/L), NH_4_^+^ (μmol/L), SiO_3_^2−^ (μmol/L), and PO_4_^3−^ (μmol/L) were determined using an QuAAtro auto-continuous flow analyzer (SEAL, Germany). In addition, the difference of environmental factors between the outer reef slope and the lagoon in HYI was tested by Welch’s *t*-test, and the statistical significance of difference between two groups was examined by the two-tailed Mann–Whitney test using IBM SPSS.v19.

### Sample collection and total holobiont DNA extraction

A total of 81 coral samples were collected from seven families, 12 genus, and 18 species with distinct thermal bleaching tolerance in the outer reef slope and lagoon of HYI; these coral species include *G. retiformis*, *A. nana*, *F. halicora*, *A. anthocercis*, *I. palifera*, *I. cuneata*, *G. pectinata*, *A. gemmifera*, *C. aspera*, *Leptoria phrygia*, *P. lutea*, *M. ampliata*, *P. woodjonesi*, *P. versipora*, *D. speciosa*, *P. verrucosa*, *Hydnophora exesa*, and *L. scutaria* (Table 1). At each coral habitat, we collected morphologically distinct colonies along linear transects of at least 10 m apart at depths ranging from 2 to 15 m at each one of the three sites, which were separated by as much as 4 km. Only adult coral colonies have been collected to control for the effect of age on microbial composition (23). Coral fragments (~2–3 cm^2^) were obtained by chisel and hammer from a depth range of 2–15 m via SCUBA diving. The coral samples were cleaned by artificial sterile seawater (salinity: 35‰) to ensure they were not disturbed with free-living Symbiodiniaceae and fungi. All fragments were transferred directly in preloaded 2-mL cryotubes containing 95% ethanol or 20% dimethyl sulfoxide buffer (145) and stored at −20°C until DNA extraction. The total holobiont DNA of 18 coral species have been extracted using the DNeasy Plant Mini Kit (Qiagen, Hilden, Germany) and DNeasy Blood and Tissue Kit (Qiagen, Hilden, Germany) and extraction processes according to the manufacturer’s instructions.

### PCR amplification, next-generation sequencing, and microbiome identification

The Symbiodiniaceae rDNA ITS2 region was amplified with the primer pair ITSintfor2 (5′-GATTGCAGAACTCCGTG-3′) (144) and ITS2-reverse (5′-GGGATCCATATGCTTAAGTTCAGCGGGT-3′) (146). The fungal ITS region was amplified with the primer pair ITS3F (5′-GCATCGATGAAGAACGCAGC-3′) and ITS4R (5′-TCCTCCGCTTATTGATATGC-3′) (147). PCR was performed with ~10 ng of DNA, 1.6 µL (5 µm) primer, 0.4 µL *Trans* Start Fastplu DNA Polymerase, 0.2 µL BSA, 4 µL 5 × FastPfu Buffer, 2 µL of 2.5 mM dNTPs, and ddH_2_O to a total volume of 20 µL. PCR amplification was conducted on an ABI GeneAmp 9700 thermocycle controller with the following program: 3 min at 95°C, followed by 35 cycles of 95°C for 30 s, 55°C for 30 s, 72°C for 45 s, and a final extension at 72°C for 10 min. The PCR products were purified using the QIAquick Gel Extraction Kit (Qiagen, Hilden, Germany), which were pair-end sequenced on an Illumina Miseq platform (Majorbio, Shanghai, China) using 2 × 300 bp mode based on standard protocols after entry quality control and adapter ligation.

### Microbiome sequence data processing and ecological indices analysis

Microbiome sequence processing was performed by Quantitative Insights Into Microbial Ecology 2 (QIIME 2) framework (148). Following the removal of primers, the forward and reverse reads were independently truncated to their appropriate lengths, then paired, dereplicated, subjected to quality control, cleaned, and finally clustered into ASV using the denoise-paired method within the DADA2 algorithm (149). For Symbiodiniaceae, the quality-filtered reads were aligned to the ITS2 database using local BLASTN, and the parameters were following the pipeline detailed by Chen et al. (85, 117). To avoid disturbance of multicopy marker and intragenomic variation of the Symbiodiniaceae ITS2 region (150, 151), we used sequence-based ITS2 (sequences were present at a minimum cut-off of >5% for at least 1 of the 81 samples) analysis to identify dominant Symbiodiniaceae sub-clades (69, 85, 117), which were recognized as biologically relevant entities of Symbiodiniaceae (121, 152). The identification of dominant Symbiodiniaceae sub-clades were used to analyze the Symbiodiniaceae community composition. In addition, the ASVs were employed for the analysis of ecological indices, including measures of alpha and beta diversity, within the Symbiodiniaceae community. However, the interpretation of individual base pair differences may introduce ambiguity to the ASVs clustered based on Symbiodiniaceae ITS sequences (153). Therefore, the ASVs of ITS2 were aligned to a non-redundant ITS2 database using local BLASTN, and non-Symbiodiniaceae ASVs were removed (85). The Symbiodiniaceae reads were uniformly rarefied to a consistent sequencing depth of 26,602 reads per sample. For ASVs of fungi, the Naïve–Bayes classifier was trained on the Unite 8.0/ITS fungi database for taxonomic assignment (154), and sequences assigned to chloroplast, mitochondria, eukaryote, and unknown at the phylum level were removed. The fungal reads were rarefied to 26,446 sequences per samples, using 1,000 iterations of random subsampling without replacement. This approach was employed to ensure that the results of fungal community ecological indices (e.g., alpha and beta diversity) can be effectively compared among samples and groups. The alpha (Chao1 richness index) and beta diversity (Bray–Curits dissimilarity) of Symbiodiniaceae and fungi communities among distinct coral species was analyzed by the Vegan package in R (155). The core ASVs of Symbiodiniaceae and fungi were identified by QIIME 2 and Venn diagram visualization (148), and ASVs consistently present in all coral species and >80% of the samples were selected as representative taxa of the core microbiome (17). The IndicSpecies was employed to identify the indicators of coral-associated Symbiodiniaceae (dominant sub-clades) and fungi (fungal ASV) using the following parameters: minimum specificity and minimum sensitivity set to 70%, *P* value < 0.05 and 1,000 permutations (156, 157). The relative abundance of indicators of Symbiodiniaceae and fungi was drawn as heat map. The phylogenetic tree was constructed by sequences of indicators of dominant Symbiodiniaceae sub-clades and potential ancestor of *Cladocopium* (C1 and C3 sub-clades) using maximum likelihood methods (60), which was aimed to speculate on the potential thermal tolerance of Symbiodiniaceae (31, 35, 69). The robustness of the tree was assessed with 1,000 bootstrap replicates. The FUNGuild tool was used to perform the prediction of fungal ecological function groups (158), and the percentages of fungal functional groups were falling into the categories of “unknown” and “known,” in addition to those known functional groups with relative abundance exceeding 1% of the known functional groups which were visualized by the ggplot2 package in R.

### Molecular ecological network constructed and topological property analysis

To explore the potential and complexed interactions between Symbiodiniaceae and fungi in coral holobiont, the SFIN was inferred by CoNet plugin in Cytoscape 3.9.1 (159, 160). Briefly, the dominant Symbiodiniaceae sub-clades and fungal ASVs were used to construct the data set, and the screening threshold has been set as taxa present in at least two samples and has more than 50 reads. The pairwise correlations among microbial taxa in different coral species were estimated by two measures of correlations (Pearson and Spearman correlations), one measure of similarities (mutual information), and two measures of dissimilarity (Bary–Curtis and Kullback–Leibler dissimilarity). Primarily, 1,000 positive and 1,000 negative edges were retrieved as thresholds for five measures, and 1,000 normalized permutations and 1,000 bootstrap scores were generated to mitigate the combinatorial bias. Brown’s method was used to calculate and merge the measure-specific *P* value (161), and the multiple comparisons was corrected by the Benjamini–Hochberg procedure (162). Moreover, only statistically significant correlations (*P* values < 0.05) were accepted for SFIN analysis. Subsequently, the visualization of SFIN was conducted with Gephi 0.9.7 and Cytoscape 3.9.1, and only co-occurrence correlations were drawn in figure.

The topological properties of SFIN were calculated using the igraph package in R, which include the average degree, network diameter, network centralization, average path length, clustering coefficient, modularity, and betweenness centrality. In general, the network diameter is defined as the indicator of the network scale (127). The average degree describes the average number of interactions per node, which can indicate the intensity of interaction among microbial taxa (125, 126). The shortest network distance between all pairs of microbial taxa was measured by average path length, which has close association with the efficiency of energy or information transmission within the network (124, 134, 163). The clustering coefficient was known as a measure of degree to which nodes in a network tend to cluster together, which suggest stability and robustness of the microbial interaction network structure (126, 127). The modularity of microbial networks was able to reflect the degree to which a network is divided into individual compartments, and nodes have strong connections with other nodes within the same module and have weak association with nodes in other modules (127, 164). Thus, the modularity was used to estimate the niche differentiation and specialization of microbial community. In addition, betweenness centrality calculates the shortest path through a microbial network and keeps record of how many times a node in a network is traversed (165), which can reflect the resilience and connectivity of microbial community (132, 135). The complexity of SFIN was determined as linkage density (links per node) among distinct coral species (130). To identify the key driver of SFIN, the percentage of the network node for Symbiodiniaceae and fungi was calculated.

### Statistical analyses

The interspecific differences of the Chao1 richness index of Symbiodiniaceae and fungal community were assessed by the Kruskal–Wallis test by performing in GraphPad Prism 8, and the Dunn test was used for *post hoc* multiple comparisons of significant Kruskal–Wallis test results. PERMANOVA was used to test the significance of interspecific differences of Symbiodiniaceae and fungal community structure with 9,999 permutation-based Bray–Curtis dissimilarity matrix. The results of PERMANOVA were visualized by the PCoA generated by the Bray–Curtis distance in the Vegan package in R (155). The SLR was used to construct the correlations between ecological indices (e.g., Chao1 richness index, abundance of pathogenic fungi, and topological parameter of SFIN) and coral thermal bleaching percentage. Pearson’s correlation was employed to calculate the coefficient of determination (*R*^2^) using GraphPad Prism 8, and the statistical significance was considered at *P* < 0.05. In addition, the difference of the percentage of network node between Symbiodiniaceae and fungi in SFIN was tested by Welch *t*-test, and the two-tailed Mann–Whitney test was conducted to examine the statistical significance of the difference between two groups using IBM SPSS.v19.

## Data Availability

The NGS raw read data set of Symbiodiniaceae ITS2 and Fungal ITS amplicons has been stored in the NCBI Sequence Read Archive database (SRA), with the accession numbers SRP432680 and SRP432640 under BioProject numbers PRJNA955669 and PRJNA955684, respectively.

## SUPPLEMENTAL MATERIAL

Table S1 and Table S2; Figure S1 aem.01939-23-s0001.pdf Environmental factors, indicative capacity values of fungi and the correlation among fungal community dissimilarity, coral thermal bleaching susceptibility and Chao1 richness index of fungi.

ASM does not own the copyrights to Supplemental Material that may be linked to, or accessed through, an article. The authors have granted ASM a non-exclusive, world-wide license to publish the Supplemental Material files. Please contact the corresponding author directly for reuse.
