# Supplementary material for: The microbiome dynamics and interaction of endosymbiotic Symbiodiniaceae and fungi are associated with thermal bleaching susceptibility of coral holobionts
Source: Appl Environ Microbiol. 2024 Mar 6;90(4):e01939-23. doi: 10.1128/aem.01939-23 (PMC11022545; doi:10.1128/aem.01939-23)
Supplement: Table S1 and Table S2; Figure S1 — Environmental factors, indicative capacity values of fungi and the correlation among fungal community dissimilarity, coral thermal bleaching susceptibility and Chao1 richness index of fungi. [file aem.01939-23-s0001.pdf]

Supporting information

The microbiome dynamics and interaction of endosymbiotic Symbiodiniaceae and fungi associate with thermal bleaching susceptibility of coral holobionts

Biao Chen [a,b], Yuxin Wei [a], Kefu Yu [a,b*], Yanting Liang [a], Xiaopeng Yu [a], Zhiheng Liao [a,c], Zhenjun Qin [a], Lijia Xu [d], Zeming Bao [a]

[a] Guangxi Laboratory on the Study of Coral Reefs in the South China Sea; Coral Reef Research Center of China; School of Marine Sciences, Guangxi University, Nanning, China

[b] Southern Marine Science and Engineering Guangdong Laboratory (Zhuhai), Zhuhai, China

[c] Key Laboratory of Environmental Change and Resource Use in Beibu Gulf, Ministry of Education, Nanning Normal University, Nanning, China

[d] South China Institute of Environmental Sciences, MEE, China

Running title: Microorganisms drive coral thermal adaptive patterns

*Corresponding Author

Prof. Kefu Yu E-mail : kefuyu@scsio.ac.cn

Table S1. Summary of the environmental factors between outer reef slope and lagoon in of coral reef in Huangyan Island.

Sampling areas	Temperature (℃)	Salinity (PSU)	DO (mg/L)	pH	Turbidity (FNU)	SiO_3^{2-} (μmol/L)	PO_4^{3-} (μmol/L)	NH_4^+ (μmol/L)	NO_3^- (μmol/L)	NO_2^- (μmol/L)
outer reef slope	31.15±0.45	34.2±0.3	5.26±0.06	8.41±0.07	0.21±0.41	2.896±0.307	0.077±0.004	0.650±0.102	0.613±0.09	0.053±0.008
lagoon	31.70±0.57	34.2±0.6	5.37±0.28	8.38±0.13	0.19±0.20	3.182±0.235	0.083±0.007	0.676±0.058	0.677±0.125	0.051±0.009

Table S2. The statistics of indicative capacity values of fungi among coral species in Huangyan Island.

	Indicative capacity values of fungi																		
**Fungal taxa**	***Goniastrea retiformis***	***Acropora nana***	***Favites halicora***	***Acropora anthocercis***	***Isopora palifera***	***Isopora cuneata***	***Goniastrea pectinata***	***Acropora gemmifera***	***Coelastrea aspera***	***Leptoria phrygia***	***Porites lutea***	***Merulina ampliata***	***Pocillopora woodjonesi***	***Plesiastrea versipora***	***Dipsastraea speciosa***	***Pocillopora verrucosa***	***Hydnophora exesa***	***Lobactis scutaria***	**p value**
**Didymellaceae**	0.000	0.302	0.013	0.000	0.035	0.036	0.000	0.000	0.000	0.000	0.000	0.000	0.000	0.000	0.000	0.000	0.000	0.000	0.043
Schizophyllum	0.314	0.000	0.000	0.000	0.000	0.059	0.000	0.000	0.039	0.039	0.000	0.000	0.000	0.000	0.000	0.000	0.000	0.000	0.035
Colletotrichum	0.000	0.000	0.333	0.000	0.000	0.000	0.000	0.000	0.000	0.000	0.000	0.000	0.000	0.000	0.000	0.000	0.000	0.000	0.040
**Chaetomiaceae**	0.000	0.000	0.000	0.000	0.000	0.000	0.000	0.000	0.000	0.500	0.000	0.000	0.000	0.000	0.000	0.000	0.000	0.000	0.017

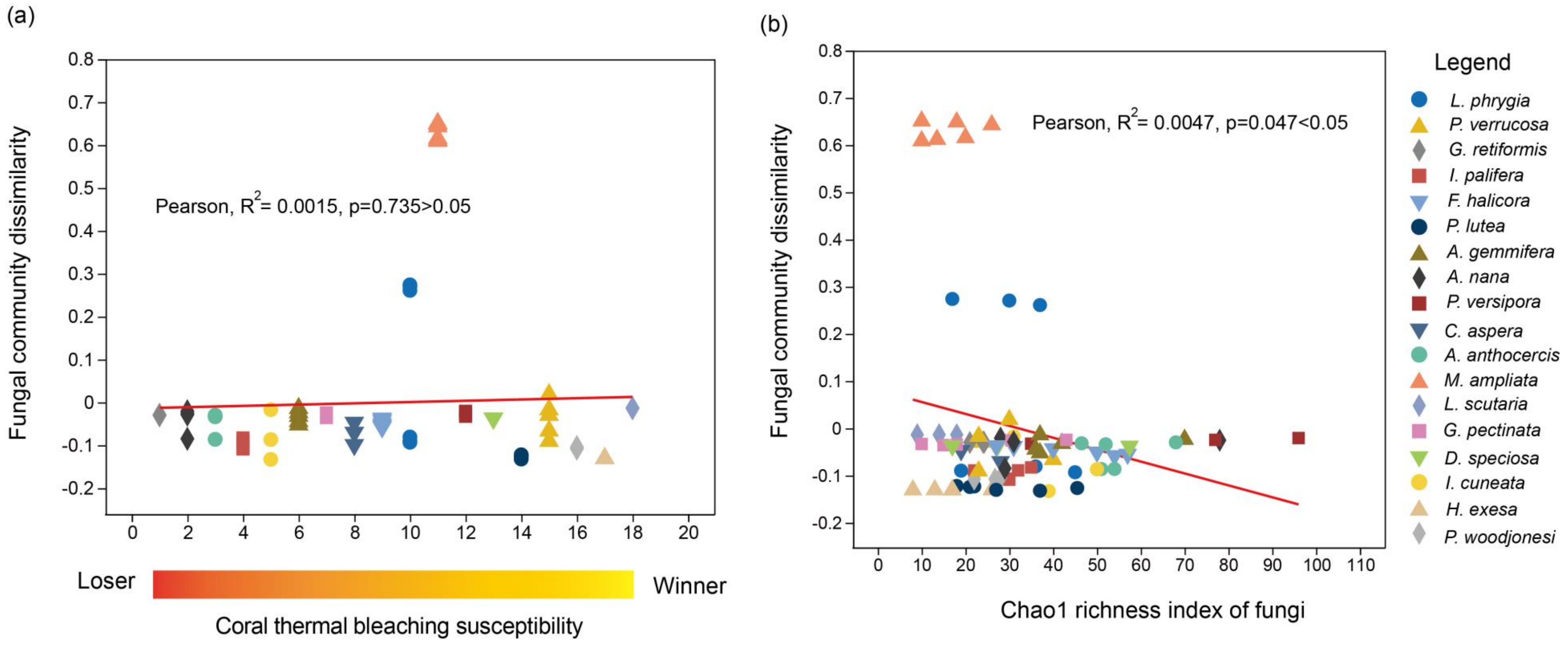

Figure S1. The correlations among fungal community dissimilarity (beta diversity), richness and coral thermal bleaching susceptibility. (a) There was a no significantly association between fungal community dissimilarity and thermal bleaching susceptibility, but the increase of Chao1 richness index will decrease the beta diversity of fungal community, significantly.
